# Expression of chemokine receptor CXCR4 in esophageal squamous cell and adenocarcinoma

**DOI:** 10.1186/1471-2407-6-290

**Published:** 2006-12-18

**Authors:** Ines Gockel, Carl C Schimanski, Christian Heinrich, T Wehler, K Frerichs, Daniel Drescher, Christian von Langsdorff, Mario Domeyer, Stefan Biesterfeld, Peter R Galle, Theodor Junginger, Markus Moehler

**Affiliations:** 1 Department of General and Abdominal Surgery, Johannes Gutenberg-University, Mainz, Germany; 2 Department of Internal Medicine, Johannes Gutenberg-University, Mainz, Germany; 3 Institute of Pathology, Johannes Gutenberg-University, Mainz, Germany

## Abstract

**Background:**

Prognosis of esophageal cancer is poor despite curative surgery. The chemokine receptor CXCR4 has been proposed to distinctly contribute to tumor growth, dissemination and local immune escape in a limited number of malignancies. The aim of our study was to evaluate the role of CXCR4 in tumor spread of esophageal cancer with a differentiated view of the two predominant histologic types – squamous cell and adenocarcinoma.

**Methods:**

Esophageal cancer tissue samples were obtained from 102 consecutive patients undergoing esophageal resection for cancer with curative intent. The LSAB+ System was used to detect the protein CXCR4. Tumor samples were classified into two groups based on the homogeneous staining intensity. A cut-off between CXCR4*w *(= weak expression) and CXCR4*s *(= strong expression) was set at 1.5 (grouped 0 – 1.5 versus 2.0 – 3). Long-term survival rates were calculated using life tables and the Kaplan-Meier method. Using the Cox's proportional hazards analysis, a model of survival prediction was established.

**Results:**

The overall expression rate for CXCR4 in esophageal squamous cell carcinoma was 94.1%. Subdividing these samples, CXCR4*w *was found in 54.9% and CXCR4*s *in 45.1%. In adenocarcinoma, an overall expression rate of 89.1% was detected with a weak intensitiy in 71.7% compared to strong staining in 29.3% (p = 0.066 squamous cell versus adenocarcinoma). The Cox's proportional hazards analysis identified the pM-category with a hazard ratio (HR) of 1.860 (95% CI: 1.014–3.414) (p = 0.045), the histologic tumor type (HR: 0.334; 95% CI: 0.180–0.618) (p = 0.0001) and the operative approach (transthoracic > transhiatal esophageal resection) (HR: 0.546; 95% CI: 0.324–0.920) (p = 0.023) as independent factors with a possible influence on the long-term prognosis in patients with esophageal carcinoma, whereas CXCR4 expression was statistically not significant (>0.05).

**Conclusion:**

Expression of the chemokine receptor CXCR4 in esophageal cancer is of major relevance in both histologic entities – squamous cell and adenocarcinoma. Though with lack of statistical significance, strong CXCR4 expression revealed a poorer long-term prognosis following curative esophagectomy in both histologic subtypes. Thus, the exact biological functions of CXCR4 in terms of tumor dissemination of esophageal cancer is yet undetermined. Inhibition of esophageal cancer progression by CXCR4 antagonists might be a promising therapeutic option in the future.

## Background

Due to a highly malignant potential for lymph node metastasis and vascular invasion, long-term prognosis of esophageal carcinoma still is – despite curative surgery – poor with 5-year survival rates of 30% reported even recently [1]. Squamous cell carcinoma and adenocarcinoma of the esophagus exhibit a different biological behaviour, pathogenesis and location, establishing the two tumor types as separate entities [2,3]. Various studies have described the histologic differentiation as an independent prognostic factor after surgical R0-resection [4,5]. Tumor spread, at least in advanced stages, in esophageal adenocarcinoma is characterized by distant metastases as compared to squamous cell carcinoma with a tendency towards local infiltrative growth [6].

Chemokines are a family of chemoattractant proteins that are classified depending on the arrangement of amino acids adjacent to conserved cysteine residues. CXCR4 is a seven-transmembrane G protein-coupled receptor and is also known as a coreceptor for HIV. SDF-1α (stem cell derived factor α), the natural ligand for CXCR4, is a member of the CXC chemokine family that has chemotactic activity for hematopoietic progenitor cells [7-10]. Thus far, chemokine signalling results in the transcription of target genes that are involved in cell invasion, motility, interactions with the extracellular matrix (ECM) and survival [11]. The expression of the chemokine receptor CXCR4 has been shown to play key mechanisms in migration and metastasis with associated tumor progression and poor prognosis in a limited number of malignancies [12-15].

The role of chemokine receptors in tumor spread of esophageal cancer with a differentiated view of the two predominant histologic types yet has to be determined.

## Methods

### Esophageal resection and tissue samples

Esophageal cancer tissue samples were obtained from 102 consecutive patients undergoing esophageal resection for cancer with curative intent at the Department of General and Abdominal Surgery, University of Mainz between 1999 and 2003. The study has been approved by the local ethics committee. Squamous cell carcinoma was diagnosed in 53 and adenocarcinoma in 49 patients. Patients with undifferentiated carcinoma or other malignant tumors of the esophagus as well as patients with neoadjuvant chemoradiotherapy were excluded from the study. Abdominothoracic esophagectomy (60.4%) was routinely performed for squamous cell carcinoma. A transhiatal procedure (37.0%) was selected for tumors with a distal location and malignancies without esophageal wall penetration, or in the presence of a high general operative risk. Transhiatal esophagectomy with abdominal and posterior mediastinal lymphadenectomy was carried out in 79.6% of adenocarcinomas, whereas the two-field procedure was done in 18.4% in the presence of advanced tumor growth or extended lymph node involvement. Histopathologic examination of the surgical specimens revealed the tumor-node metastasis classification of the International Union Against Cancer [16]. pM1 patients were defined as positive lymph node metastases at the celiac trunk (pM1 lymph). These lymph node metastases were cleared in all cases with a consecutive R0 resection.

### Immunohistochemical staining

The LSAB+ System from DakoCytomation (K0690; DakoCytomation Inc., California, USA) was used to detect the protein CXCR-4 (anti-CXCR-4, CIO115, dilution 1:200; Capralogics, USA). In brief, samples were exposed to 70°C for one hour in a humified oven and hereafter deparaffinised. After pre-incubation with hydrogen peroxide (3%) for 5 minutes and consecutive incubation with human fresh frozen plasma for one hour, the primary antibodies were applied for 2 hours at room temperature. After incubation with the secondary antibody (pooled swine-anti-goat, -anti-mouse, -anti-rabbit-antibody; LSAB+ Kit) for 15 minutes, the samples were exposed to streptavidinperoxidase for another 15 minutes and chromogen-solution (LSAB+ solution) for 15 minutes (LSAB+ Kit, respectively). Counterstaining was performed with haematoxylin (Sigma, Germany). For negative controls only the secondary antibody was used. A negative control was performed for each esophageal cancer sample. For positive controls, formalin-fixed and paraffin-embedded tissue samples of the human spleen were applied.

### Evaluation of Immunostaining

Immunostaining was evaluated by three authors independently, blinded to the patients' clinicopathological features and long-term survival. The immunohistochemical staining was analyzed according to a scoring method previously validated. Tumor samples were classified into two groups based on the homogeneous staining intensity: 0 = absent, 1 = weak, 2 = intermediate, 3 = strong staining. In the case of heterogeneous staining within the sample, the respective 0.5 points higher score was chosen, if more than 50% of cells revealed the higher staining intensity. If evaluations did not agree, specimens were reevaluated and re-classified according to the assessment given most frequently by the observers. A cut-off between CXCR4*w *(= weak expression) and CXCR4*s *(= strong expression) was set at 1.5 (grouped 0 – 1.5 versus 2.0 – 3).

### Statistical analysis

The SPSS 12.0 software package was used for statistical data analysis (SPSS, Chicago, IL, USA: 2005). Clinical data were prospectively collected in a database established for internal quality control and retrospectively analysed. The presented data are expressed as mean values (+/- standard deviation). In the comparative analysis of the different parameters between the two CXCR4 expression groups, the χ^2 ^test with Pearson's correction with cross-table calculations, or the Fisher's exact test was used for categorical parameters. The Mann-Whitney U-test served as the non-parametric method for quantitative variables. Long-term survival data were finally recorded in December 2005 and collected by telephone interview with the patients or their primary physicians. Survival probabilities were estimated using life tables and the method of Kaplan and Meier, and a log-rank analysis was carried out to determine significant differences between the patient groups. The Cox's univariate regression model was used to analyse the influence of possible prognostic factors on survival separately. Multivariate analysis of significant factors was performed using Cox's proportional hazards model. A p-value of <0.05 was considered statistically significant for all procedures.

## Results

### Patterns of CXCR4 expression in squamous cell carcinoma (SCC) and adenocarcinoma (ADC) of the esophagus

**Figure 1:**
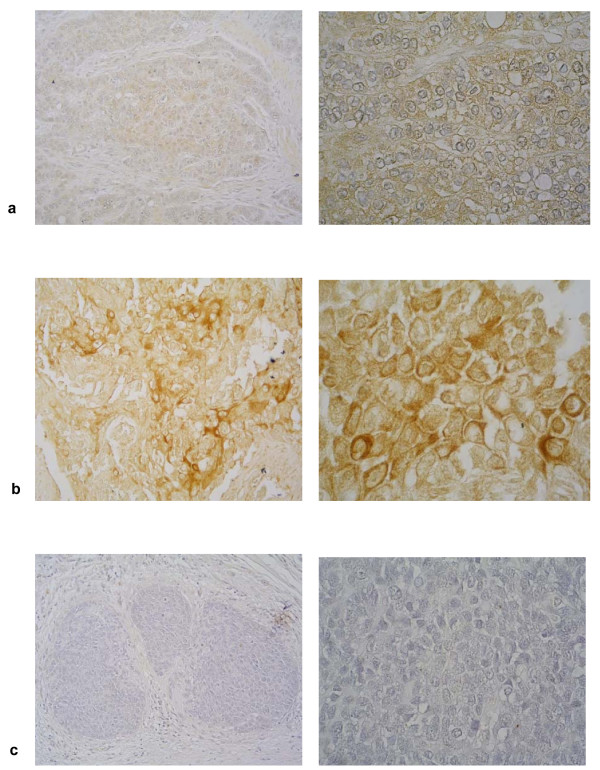
**a-c**. CXCR4-expression in squamous cell carcinoma with weak (CXCR4*w*) (a) and strong (CXCR4*s*) (b) staining intensity compared to negative control (c).

**Figure 2:**
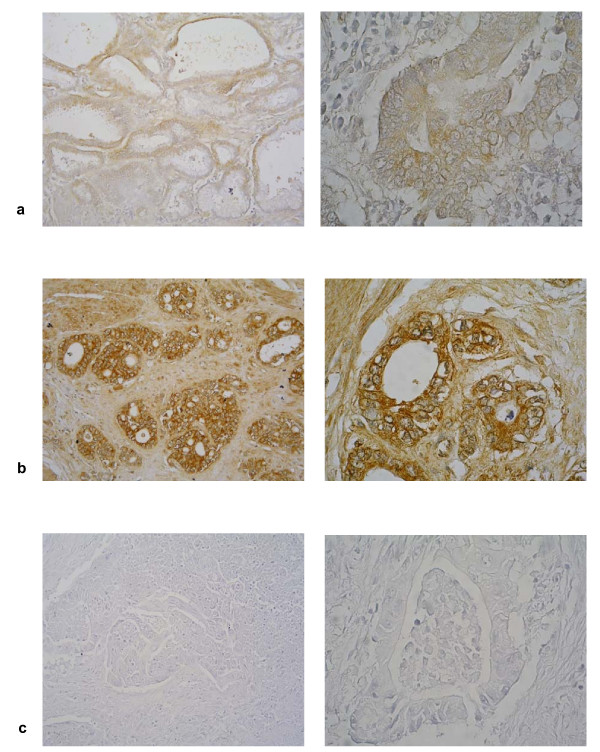
**a-c**. CXCR4-expression in adenocarcinoma with weak (CXCR4*w*) (a) and strong (CXCR4*s*) (b) staining intensity compared to negative control (c).

Staining for CXCR4 revealed predominantly a cytoplasmatic, and in a few specimens a weak membranous location of CXCR4. The respective overall expression rate for CXCR4 in squamous cell carcinoma was 94.1%. Subdividing these samples according to the previous classification, CXCR4*w *was found in 54.9% and CXCR4*s *in 45.1% (Figure 1a–c). In adenocarcinoma, an overall expression rate of 89.1% was detected with a weak intensitiy in 71.7% compared to strong staining in 29.3% (Figure 2a–c). There was no statistical significant difference in the intensity patterns of chemokine receptor expression between the two histologic tumor types (p = 0.066).

### Clinical features and CXCR4 expression

There were no significant differences between the two classes of CXCR4 expression (CXCR4*w *versus CXCR4*s*) in squamous cell carcinoma as well as in adenocarcinoma with regard to age, gender ASA-classification and tumor location (Table 1)

### Histopathologic characteristics and CXCR4 expression

In patients with squamous cell carcinoma, a significant difference between the two groups of CXCR4 expression was found for tumor grading (p = 0.030). Differences in CXCR4 intensity (CXCR4*w *versus CXCR4*s*) were not significant reflecting the T-, N-, and M-category (Table 2). Other parameters included in the analysis – separately for both tumor types – as tumor size, UICC-classification, lymphangiosis carcinomatosa and lymph node ratio (involved lymph nodes × 100/dissected lymph nodes) revealed no significant differences in CXCR4 expression.

### Long-term survival and CXCR4 expression

**Figure 3:**
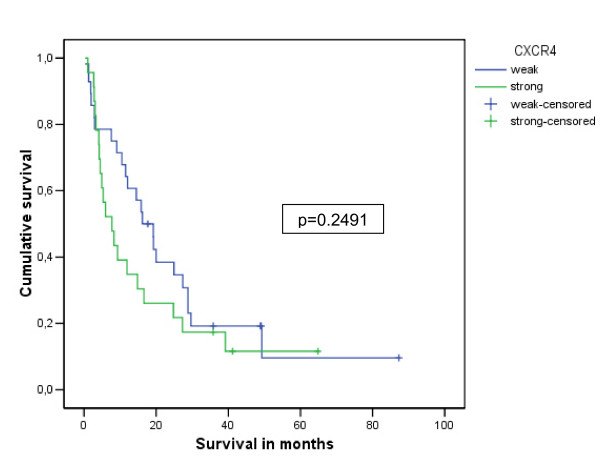
Kaplan-Meier survival curves for patients with squamous cell carcinoma and weak (CXCR4*w*) versus strong (CXCR4*s*) chemokine receptor expression.

**Figure 4:**
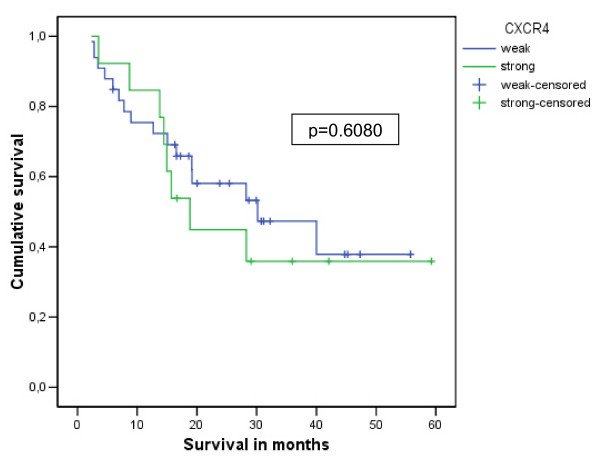
Kaplan-Meier survival curves for patients with adenocarcinoma and weak (CXCR4*w*) versus strong (CXCR4*s*) chemokine receptor expression.

In squamous cell carcinoma, patients with a weak CXCR4 expression had a mean survival of 25 (+/- 5 SD) months after surgery as compared to only 17 (+/- 4 SD) months in the group with strong chemokine expression (Figure 3). However, this difference was statistically not significant (log-rank test: p = 0.2491). Patients with adenocarcinoma and CXCR4*w *had a mean survival of 32 (+/- 4 SD) months while the CXCR4*s *group did not significantly differ in prognosis with 31 (+/- 6 SD) months after esophageal resection (log-rank test: p = 0.6080) (Figure 4).

For multivariate analysis, a model of prediction of survival time was established using the Cox's proportional hazards analysis. Variables selected for this model were: CXCR4 expression, pT-, pN-, pM-category, histologic tumor type, tumor grading and the operative procedure (transthoracic versus transhiatal esophageal resection). The analysis identified the pM-category with a hazard ratio (HR) of 1.860 (95% CI: 1.014–3.414) (p = 0.045), the histologic tumor type (HR: 0.334; 95% CI: 0.180–0.618) (p = 0.0001) and the operative approach (HR: 0.546; 95% CI: 0.324–0.920) (p = 0.023) as independent factors with a possible influence on the long-term prognosis in patients with esophageal carcinoma (Table 3).

## Discussion

CXCR4 expression was detected in both types of esophageal cancer – squamous cell and adenocarcinoma. A slightly higher expression of CXCR4 (94.1% in squamous cell carcinoma and 89.1% in adenocarcinoma of the esophagus) was found as compared to other studies. Our data provide evidence that expression of CXCR4 by primary tumor cells is associated with malignant transformation in esophageal cancer, consistent with recent findings by Kaifi et al [17], but contrasted to a preceding investigation of Mitra et al., who used reverse transcriptase-polymerase chain reaction [18]. The only study investigating CXCR4 expression in esophageal squamous cell *and *adenocarcinoma of the esophagus before- to our knowledge – has been published by Kaifi et al [17]. Thus, the difference in the tumor-biologic behaviour of the two esophageal cancer types with a high incidence rate of locoregional spread in squamous cell carcinoma as compared to the development of distant metastasis in adenocarcinoma is not yet fully understood.

Although the exact mechanism of CXCR4 activity has not thoroughly been elucidated, it may induce or support carcinogenesis through the interaction of CXCR4 with its ligand SDF-1α, which mediates the activation of phosphatidylinositol 3-kinase and Akt, resulting in cell proliferation [19,20]. Interestingly, CXCR4 is regulated by different external factors, such as hypoxia (hif-1- pathway) and the activation of adenosine-receptors, as well as by internal alterations as the inactivation of tumor suppressor genes pVHL, p53, over-expression of NFkB and DNA methylation [21-25]. Homing factors, inducing chemotaxis to target organs of dissemination, have been proposed as the major inductor of tumour cell dissemination and metastatic growth, as the filter theory does not sufficiently explain the growth of metastases in target organs [26-28]. SDF-1α, which is produced elsewhere, is most intense in typical "homing organs" such as lungs, bone marrow, liver and lymph-nodes compared with other non-homing tissues [14,17]. The interaction between esophageal cancer-expressed CXCR4 and SDF-1α may be a key event in directing malignant cells to these "homing organs", and this mechanism may also account for metastasis from other organs, as previously reported [29-31]. This homing theory is underlined by the findings, that the presence of micrometastatic tumor cells in lymph nodes and bone marrow has been associated with poorer survival [32,33].

In our study, the CXCR4 expression profile did not differ significantly between squamous cell carcinoma and adenocarcinoma in esophageal cancer, proposing additional factors responsible for their different patterns of tumor spread. It may also support the hypothesis of a similar pathway of CXCR4's influence on the lymphatic and hematogeneous tumor cell dissemination. Although the interaction of CXCR4 with the lymphatic system has not been investigated as intense as the hematogeneous dissemination, CXCR4 inhibition resulted in suppression of breast cancer lymph node metastases, implying common routes in both systems [34].

In both histologic types, strong CXCR4 expression revealed a poorer long-term prognosis following esophagectomy in curative intent. Thus, CXCR4 was not significantly associated with survival in contrast to a recent study by Kaifi et al, published in the *Journal of the National Cancer Institute, 2005*. Though a lack of statistical significance, there was a trend to a less favourable outcome associated with an increased intensity of CXCR4, as has previously been demonstrated for other malignancies [12-15]. The histologic tumor type in the Cox regression analysis was statistically significant (p = 0.0001), which might be explained by the different biological behaviour associated with squamous cell and adenocarcinoma as published before (Gockel I, et al. World J Surg 2006;30:183–190) [6]. Apart from the histologic tumor type, the pM-category proved an independent prognostic factor using Cox's proportional hazards model. Although all patients with pM1 (lymph) (= celiac trunk positive lymph node metastases) had undergone curative resection, this emphasizes the impact of systemic disease on impaired survival. The surgical procedure was another significant parameter in this analysis with a more favourable outcome following the transthoracic esophagectomy with extended lymphadenectomy, at least in patients with squamous cell carcinoma, as recently published [35].

## Conclusion

Expression of the chemokine receptor CXCR4 in esophageal cancer is of major relevance in both histologic entities – squamous cell and adenocarcinoma. Though with lack of statistical significance, strong CXCR4 expression revealed a poorer long-term prognosis following curative esophagectomy in both histologic subtypes. The type of the surgical resection in esophageal cancer remained one of the most important factors with an influence on the patients' long-term prognosis.

Thus, the exact biological functions of CXCR4 in terms of tumor dissemination of esophageal cancer is yet undetermined. Inhibition of esophageal cancer progression by CXCR4 antagonists might be a promising therapeutic option in the future.

## Competing interests

The author(s) declare that they have no competing interests.

## Authors' contributions

I.G., C.C.S. and M.M. initiated the presented study, participated in its design and coordination and carried out the study. Before this specific study, C.C.S. and M.M. initiated other translational research projects on CXCR4 at the University of Mainz. C.H., T.W., D.D., C.v.L. and K.F. carried out the study. S.B. performed the histopathologic evaluation. M.D. carried out the statistical analysis. P.R.G. and T.J. participated in the coordination of the study. I.G. and C.C.S. drafted the first version of the manuscript and all authors read and approved the final manuscript.

## Pre-publication history

The pre-publication history for this paper can be accessed here:



## Figures and Tables

**Table 1:** Clinical features and CXCR4-expression.

	**SCC**			**ADC**		
	**CXCR4*w***	**CXCR4*s***	**p-value**	**CXCR4*w***	**CXCR4*s***	**p-value**
age (years)	56.8 (+/- 7.5 SD)	58.4 (+/- 8.5 SD)	0.501	62.0 (+/-10.3 SD)	59.3 (+/- 6.9 SD)	0.131
gender (% males)	89.3	87.0	0.566	87.9	92.3	0.561
ASA (%)						
-I	32.1	30.4	0.973	69.7	46.2	0.067
-II	60.7	65.3		27.3	46.2	
-III	7.2	4.3		3.0	7.6	
tumor location (%)						
-proximal 1/3	10.7	13.0	0.712	0	0	0.499
-middle 1/3	50.0	52.2		3.0	7.7	
-distal 1/3	39.3	34.8		97.0	92.3	

**Table 2:** Histopathologic characteristics and CXCR4-expression.

	**SCC**			**ADC**		
	**CXCR4*w***	**CXCR4*s***	**p-value**	**CXCR4*w***	**CXCR4*s***	**p-value**
pT-category						
-pT1-2	17.9	21.7	0.857	48.5	38.5	0.856
-pT3-4	82.1	78.3		51.5	61.5	
pN-category						
-pN0	46.4	34.8	0.290	21.2	15.4	0.845
-pN+	53.6	65.2		78.8	84.6	
pM-category						
-pM0	85.7	73.9	0.261	69.7	84.6	0.259
-pM+	14.3	26.1		30.3	15.4	
tumor grading						
-G1-2	32.1	65.2	0.030*	27.3	46.2	0.315
-G3-4	67.9	34.8		72.7	53.8	

*statistically significant

**Table 3:** Cox's proportional hazards analysis.

**Variable**	**hazard ratio (HR)**	**95%-confidence interval (CI)**		**p-value**
		**lower border**	**upper border**	
CXCR4	1.472	0.836	2.593	0.181
pN-category	1.808	0.966	3.386	0.064
tumor grading	1.701	0.923	3.137	0.089
**operative approach**	**0.546**	**0.324**	**0.920**	0.023*
pT-category	1.700	0.867	3.332	0.122
**pM-category**	**1.860**	**1.014**	**3.414**	0.045*
**tumor type**	**0.334**	**0.180**	**0.618**	0.0001*

*statistically significant
